# Who is captain of the ship? Navigating the birth voyage together

**DOI:** 10.1111/aogs.15138

**Published:** 2025-04-23

**Authors:** Julia Savchenko, Andrew Kotaska

**Affiliations:** ^1^ Department of Obstetrics and Gynecology Stockholm South General Hospital (Södersjukhuset) Stockholm Sweden; ^2^ Department of Clinical Science and Education Stockholm South General Hospital (Södersjukhuset), Karolinska Institutet Stockholm Sweden; ^3^ Department of Obstetrics, Gynecology, and Reproductive Sciences, Faculty of Health Sciences University of Manitoba Winnipeg Manitoba Canada; ^4^ School of Population and Public Health University of British Columbia Vancouver British Columbia Canada

Times are changing. According to the WHO, the clinical management of labor and childbirth is well understood, while the emotional and psychological needs of women giving birth are now a priority.^1^ This emphasis on the subjective experience of childbirth is relatively new. For generations, obstetricians were busy delivering babies, stopping bleeding, preventing convulsions, and treating childbed fever—working hard to ensure that a mother and her newborn survived without serious damage or disability. The physical well‐being of mother and child is still a priority; however, medical safety alone is no longer enough: other aspects of the birth experience appear to be at least as important to families.

*Feruza wants her real name to appear in this editorial. When one of the authors (J.S.) met her, she was 40 years old, 157 cm tall, para 3, and 36 weeks pregnant with dichorionic twins. The presenting twin was breech and small for gestational age. Her first birth was an elective cesarean for breech, followed by two fast, normal vaginal births. In her current pregnancy, she was repeatedly advised to undergo an elective cesarean section. She eventually responded by missing her booked antenatal visits and not answering phone calls.*

Within the profession, attitudes toward the shift in focus from objectively measurable outcomes to childbirth experience are mixed. There is reason to be proud of this development—it reflects that we are winning the battle for maternal and perinatal health. Unfortunately, serious complications still occur, but they are no longer the everyday fear they once were. Instead, they are seen as rare, catastrophic events that are not expected to happen. Families often believe that childbirth should be a pleasant life event; and obstetricians and midwives sometimes struggle when finding themselves working in an “experience industry” rather than “simply” saving lives. In a sense, obstetrics has become a victim of its own success—expectations are high and meeting them is not always easy.

*Feruza clearly knew what she wanted. Her elective cesarean was tough for her physically and mentally, but she was very happy with her two vaginal births. She received abundant information about the risks of breech birth, growth restriction, uterine rupture, placental abruption, cord prolapse, birth asphyxia, and interlocked twins; yet she remained completely confident that she could give birth naturally and that everything would be fine. She definitively refused a planned cesarean, and when asked if she would accept an emergency cesarean if indicated in labor, she responded that she did not think it would be necessary.*

It does not require a medically adverse outcome to feel dissatisfied. Discussion around obstetric violence, disrespectful care, coercion, and physical and psychological abuse during childbirth is growing.^2^, ^3^, ^4^ Having this conversation is valuable, if difficult, and important to handle with care. “Whose fault is it when things go wrong or feel wrong?” is a natural question, but often not constructive. It is easy to feel unfairly accused and become defensive: after all, we're all trying to do our best, right? Or to simply provide information and “wash our hands” of the situation, placing responsibility for negative outcomes on the patient, who claims to be willing to accept the risks. The challenge is building up a relationship of trust and reaching a shared understanding that will optimize safety and experience.

*Inferring specific risks in individual cases from a general body of evidence is tricky; and it is difficult to understand, explain, and compare complex risks*.^5^, ^6^, ^7^ *In Feruza's case, when would the risk of antepartum stillbirth and placenta abruption outweigh the risk of labor induction with a breech presenting twin? Should induction begin with amniotomy or oxytocin with intact membranes? Would neuraxial anesthesia be beneficial or harmful (if she would agree to it)? Which rescue maneuvers would be most effective in the event of locked twins, and how should we prepare the team? After extensive consultation—within the clinic, regionally, and internationally, we suggested careful labor induction at 38 weeks' gestation, which Feruza eventually accepted.*

In general, patients have the right to refuse treatment but cannot demand it. However, the conflict between childbirth care professionals and mothers, individual or collective, can be exhausting and counterproductive for both parties. Finding common ground is mutually beneficial. It requires respect, curiosity, competence, and sometimes clinical courage to meet women where they are—wherever that may be—and to help them get to where they want to go. In most cases, this is possible, but only through teamwork: each team member contributing their expertise and effort, acting together, always remembering that the mother is the captain.

*We alerted the neonatologists, operating room staff, and anesthesiologists. After membrane sweeping, regular contraction quickly began, and Feruza's cervix dilated from 4 to 6 cm. Then progress stopped. We offered amniotomy, oxytocin, and cesarean again, but none were accepted. Feruza alternated between active movement in a variety of upright positions and periods of rest. After several hours in labor, she suddenly accepted our recommendation for cesarean on her terms: no cord clamping, immediate skin‐to‐skin contact for at least 2 h, and no separation from the babies unless medically necessary. The cesarean was uneventful, and two healthy babies were born with normal Apgar scores and cord gases.*

To promote understanding between birth care providers and families, we suggest:

- *Nuanced discussion*. In order to provide mothers with as objective information about risks and benefits as possible, we have a duty to interpret complex clinical situations. Limited evidence and maternal choices outside mainstream practice can make this challenging. Removing our emotions from the equation and keeping maternal values at the forefront helps build a therapeutic alliance, trust, and a maternal sense of control.^8^
- *Clear concepts of harm*. The term “obstetric violence” should be reserved for forceful intervention directly against a woman's wishes or with intent to harm. This is different from “disrespectful care,” which includes dismissive attitudes and lack of empathy or support, sometimes obvious, sometimes more subjective and difficult to define. “Substandard care”—the failure to apply the standard of care—is different again. Obstetrical violence may involve substandard care and is always disrespectful, but the reverse is not true. All three cause harm and must be avoided; however, discussion and collaboration around complex issues is aided if the concepts are not confused.^9^
- *Positive focus*. It is helpful to move beyond what was done wrong and by whom to explore what can be improved and how. Concepts such as autonomy, empowerment, shared decision‐making, support, resilience, healing, and a sense of accomplishment should be emphasized, and the sharing of positive experiences encouraged.^8^, ^10^
- *Acknowledgement that adverse events may happen*. A zero‐risk vision of childbirth safety is unrealistic and may be harmful, potentially leading to “defensive obstetrics” and unnecessary interventions. Some incidents are beyond our control or arise from prioritizing patient autonomy.^11^ Caregivers must feel secure in supporting patients' goals without fear of punitive measures, and they should have access to help as “second victims” if needed.^12^

*While Feruza appeared to be disappointed while being rolled into the operating room, she later rated her birth experience as 10 out of 10, saying she cried tears of joy when lying with her two newborns on her chest, with placentas still attached in a bowl nearby (not our usual practice). As for the obstetric team, the dominant feeling was perhaps relief, but also satisfaction.*

Times are changing, and so are we. While there is still much about the clinical management of labor to understand, support for autonomy, shared decision‐making, and respectful birth care is becoming the norm. Every woman is and has to be seen as the master of her vessel. The ultimate authority and responsibility lie with her. We are her pilot and crew—providing in‐depth knowledge of local waters, advice to avoid hazards, and skills to navigate tricky passages and help if her ship runs aground. Women want to feel in control and cared for. Professionals want to be trusted and to apply the best of their knowledge and expertise. These desires are synergistic, not opposing. We share a common goal—to reach harbor, as safely and happily as possible.

## ETHICS STATEMENT

Feruza gave her consent for her birth story to be published.

## Data Availability

Data sharing is not applicable to this article as no new data were created or analyzed in this study.
